# Genome-Wide Association Study Identifies New Candidate Markers for Somatic Cells Score in a Local Dairy Sheep

**DOI:** 10.3389/fgene.2021.643531

**Published:** 2021-03-22

**Authors:** Anna Maria Sutera, Angelo Moscarelli, Salvatore Mastrangelo, Maria Teresa Sardina, Rosalia Di Gerlando, Baldassare Portolano, Marco Tolone

**Affiliations:** ^1^Dipartimento Scienze Veterinarie, University of Messina, Messina, Italy; ^2^Dipartimento di Scienze Agrarie Alimentari e Forestali, University of Palermo, Palermo, Italy

**Keywords:** mastitis, local dairy sheep, GWAS, SNPs arrays, candidate genes

## Abstract

In the Mediterranean basin countries, the dairy sheep production is usually based on local breeds, which are very well-adapted to their production systems and environments and can indeed guarantee income, employment, and economic viability in areas where production alternatives are scarce or non-existent. Mastitis is still one of the greatest problems affecting commercial milk production. However, genetic evaluation of mastitis is particularly difficult because of its low heritability and the categorical nature of the trait. The aim of this study was to identify genomic regions putatively associated with somatic cells count (SCC) in the local economically important Valle del Belice sheep breed using of deregressed breeding values (DEBV) as response variables. All the samples were genotyped using the Illumina OvineSNP50K BeadChip. Genome-wide association analysis was carried out based on regression of DEBV. A total of eight markers were found to be significantly associated with log-transformed SCC. Several candidate genes associated with SCC were identified related to immunity system and udder conformation. The results can help improving the competitiveness of the local Valle del Belìce breed. Further studies considering a higher sample size or independent population will be needed to confirm our results.

## Introduction

In the Mediterranean basin countries, the dairy sheep production is usually based on local breeds, which are very well-adapted to their production systems and environments and can indeed guarantee income, employment, and economic viability in areas where production alternatives are scarce or non-existent. Mastitis is the most important problem for the milk industry due to the decrease quality of milk and increased cost of flock regeneration due to early culling of ewes. It can be induced, for example, by a lack of hygiene, by pushed manual milking or feed disorder. In dairy sheep, generally, the most important agents involved in mastitis are the bacterial infections, and the most frequently isolated pathogens are coagulase-negative staphylococci (CNS) that are present on and around the udder skin (Leitner et al., 2008) with a different pathogenicity, causing clinical, and subclinical mastitis (Contreras et al., 2007; Riggio and Portolano, 2015). The udder infection determines the increase of the somatic cell count (SCC) in milk (Raynal-Ljutovac et al., 2007; Leitner et al., 2008) that causes significant damage of curd and cheese yields. Since the heritability of mastitis is low, genetic selection to improve mastitis by traditional selection is not very effective. SCC or log transformed SCC (i.e., somatic cell score, SCS) have relatively higher heritability compared to mastitis and is used as the first trait to improve mastitis resistance (Shook and Schutz, 1994). Kelly et al. (2000) found that an elevated SCC can alter the protein fractions distribution; decrease casein and lactose levels in milk; increase rennet clotting time, cheese moisture, and losses of fat and proteins in whey, and reduce curd firmness and cheese yielding. A study conducted by Sutera et al. (2018) confirmed that high levels of SCC in sheep milk are associated with milk yield losses and variations of fat and protein percentages. The estimated losses in milk yield ranged from 883 g for SCC ≤ 2,000 × 10^3^ to 1,052 g for SCC ≤ 500 × 10^3^ with an overall decrease of 16%, whereas fat and protein percentages increased to 0.06 and 0.29%, respectively. The negative effects of mastitis are provoked by a combination of animal characteristics (age, lactation stage, etc.), genetic (breed, inbreeding, etc.) and environmental factors (season, management, etc.) (Oget et al., 2019). Therefore, different individuals may have a different susceptibility to the disease, depending on their genetic heritage. In fact, there are several studies about the mastitis in dairy sheep confirming a genetic basis for mastitis resistance (Tolone et al., 2013; Oget et al., 2019), but no assumption had been made about the genes and the relative mechanisms.

The emergence of high-throughput genotyping technologies allowed routine genome-wide association studies (GWAS) to be performed in livestock populations. GWAS allows screening of the genome utilizing a large number of genetic markers spread across the entire genome to detect genetic variants associated with a particular disease or trait. The estimated breeding values (EBVs) were generally used to perform the GWAS. As an alternative, the EBVs can be “deregressed” (Garrick et al., 2009; Ostersen et al., 2011) to standardize the variance and influence of the individuals’ EBVs while still accounting for information from relatives. The use of deregressed EBVs (DEBVs) as dependent variables can improve the power of GWAS (Sell-Kubiak et al., 2015; Sevillano et al., 2015). An advantage of GWAS is that we can overcome the candidate gene approach through which sometimes significant results were not obtained due to the wrong or incomplete choice of candidate genes. In the last decades, several GWASs were conducted in sheep for milk production related traits (Sutera et al., 2019; Li et al., 2020), for fatty acids profile (Rovadoscki et al., 2018), for body weight (Ghasemi et al., 2019; Tao et al., 2020), for wool production (Wang et al., 2014), for nematode resistance (Becker et al., 2020) and ovine lentivirus resistance (White et al., 2012). To date, few GWASs have been conducted for SCC or SCS in dairy sheep (Oget et al., 2019), especially in local adapted breeds.

In Sicily, dairy sheep production represents an important resource for the local economy, and the Valle del Belice is the main local breed reared on the island for the production of traditional raw milk cheeses, at farm level by small local dairies. The breed is subjected to limited breeding selection programs for milk production traits, but shows excellent adaptability to local environments, sometimes with harsh conditions (Mastrangelo et al., 2017). Therefore, the aim of this study was to identify the genomic regions putatively associated with SCC in Valle del Belice sheep breed using of DEBVs as response variables.

## Materials and Methods

### Data and Estimation of Breeding Value

Between 2006 and 2016 the University of Palermo collected phenotypic data from 15 Valle del Belìce flocks, for a total of 1,813 individuals. The milk samples were collected aseptically from each individual from the two udder halves in sterile containers following an A4 recording procedure (ICAR, 2014), stored at 4°C and transferred to the laboratory to determine daily SCC using Fossomatic 6000 (Foss Electric Hillerød, Denmark) equipment. The phenotypic data set originated by these sampling works was composed of 15,008 observations. Animals with less than 3 test-day measurements within lactation were discarded. For each individual the following information were registered: order of parity, number of born lambs, lactation days, age, birth season and somatic cell count. Birth season was classified in three classes: 1 if the lambing was from August to November; 2 from December to March; 3 from April to July. SCC was normalized through a logarithmic transformation into somatic cell score (SCS) according to the formula of Ali and Shook (1980):

$$SCS=log_{2}\left(\frac{SCC}{100,000}\right)+3$$

Preliminary analyses using the general linear model of ASReml R (Butler et al., 2009) were performed to determine the significance of the fixed effects where the Wald tests are implemented in the form of the ANOVA method. A single trait repeatability test day (TD) animal model was performed to estimate the breeding values (EBV) as follows:

$$y=X\beta +Z_{htd}+Z_{a}+Z_{pe}+e$$

where y is the observation vector for SCS TD; β is the vector of fixed effects that includes order of parity (op: 4 classes), age at first lambing (age: 4 classes, 1 when first lambing occurred at 10–14 months of age, 2 at 15–19 months of age, 3 at 20–24 months of age, and 4 at 25–29 months of age); birth season (bs: 3 classes), interaction between herd and birth season (hbs: 74 classes) and days of lactation (dim) modeled with a Legendre polynomial of order three. Htd is the vector of interaction between herd and test day random effect; a is the vector of direct additive genetic effects (breeding values); pe is the vectors of permanent environmental effect between lactations; e is the residual vector. X and Z are the corresponding incidence matrices relating records to fixed, animal, and permanent environmental between lactations effects, respectively. The pedigree file included 5,534 animals with 178 sires and 2,548 dams. The assumptions regarding the components of the model were:

$$E\left[\begin{array}{c} y\\ b\\ htd\\ a\\ pe\\ e \end{array}\right]=\left[\begin{array}{c} Xb\\ 0\\ 0\\ 0\\ 0\\ 0 \end{array}\right]$$

and $V_{a}=A\sigma_{a}^{2}$; $V_{htd}=I\sigma_{htd}^{2}$; $V_{pe}=I\sigma_{pe}^{2}$; $V_{e}=I\sigma_{e}^{2}$ where A is the numerator relationship matrix based on pedigree and I are the identity matrix with orders equal to numbers of dams for htd and pe effects and equal to the records for residuals e. Variance components and breeding values for SCS were estimated based on REML method using ASReml R (Butler et al., 2009). In addition, EBVs were also deregressed according to Garrick et al. (2009) as follows:

$$DEBV=EBV/r^{2}$$

where, EBV is the estimated breeding value and r^2^ is the reliability of that EBV.

### Blood Sampling and DNA Extraction

A total of 476 sheep of Valle del Belice breed were sampled. About 10 mL of blood was collected from the jugular vein using vacutainer tubes containing EDTA as anticoagulant. The procedures involving animal sample collection followed the recommendation of directive 2010/63/EU. Sampling was carried out by trained veterinarians within the frame of vaccination Campaigns, hence no permission from the animal research ethics committee was necessary. Veterinarians adhered to standard procedures and relevant national guidelines to ensure appropriate animal care. Genomic DNA was extracted from each blood sample with a salting-out method (Miller et al., 1988). The DNA sample was quantified with a NanoDropND-1000 spectrophotometer (NanoDropTechnologies, Wilmington, DE, United States), diluted to a final concentration of 50 ng/mL (as required by the Illumina Infinium protocol), and stored at 4°C until use.

### Genotyping and Quality Control

All the samples were genotyped using the Illumina OvineSNP50K BeadChip v2. Position and chromosomal coordinates for each SNP were obtained from the ovine genome sequence assembly (Oar 4.0)^1^. Quality control and association analyses were performed using GenABEL package (Aulchenko et al., 2007) in R environment^2^. Only SNPs located on autosomes were extracted and considered for further analyses. Animals and markers that fulfilled the following criteria were kept in the analysis: (i) call rate per individuals and per SNPs > 95%; (ii) minor allele frequency > 2%; (iii) no extreme deviation from Hardy-Weinberg equilibrium (*P* < 10^–6^).

### GWAS Analyses

Genome-wide association analysis was carried out based on regression of DEBV with the genotypes of animals for one SNP at a time. We used the three-step approach referred to as genomic GRAMMAR-GC (Amin et al., 2007; Aulchenko et al., 2007). The advantage of this approach, especially in livestock, is that it accounts for cryptic population structure caused by the presence of closely related animals (Aulchenko et al., 2007) inferring relationships through genomic marker data. After Bonferroni correction, significant thresholds were *P* < 1.34 × 10^–6^ for genome-wide (*P* < 0.05) and *P* < 2.69 × 10^–5^ for suggestive (*P* < 0.10) (i.e., one false positive for genome scan), corresponding to −log10(P) equal to 5.87 and 4.56, respectively. Quantile-quantile (Q-Q) plots were used to analyze the extent to which the observed distribution of the statistic test followed the expected (null) distribution, in order to assess potential systematic bias due to population structure or analytical approach. Population substructure was explored using classical multidimensional scaling (MDS) in order to verify the genetic homogeneity of the sample before analysis using PLINK v1.9 (Purcell et al., 2007). The least square means of DEBV for the three genotypes affecting somatic cell count of significant SNP were also calculated by a general linear model (GLM) using R package lsmeans (Lenth and Lenth, 2018) and the significant threshold was set at *P* < 0.05.

### Annotation

Genomic regions showing significant results were further explored to identify candidate genes underlying the loci. In particular, the gene contents located at ±250 kb distances from the significant SNP were annotated using Genome Data Viewer genome browser at the National Center for Biotechnology Information Database^3^. The presence of Quantitative Traits Loci (QTLs)^4^ related with the considered trait was also checked. Finally, to investigate the biological function and the phenotypes that are known to be affected by each annotated gene, we conducted a comprehensive literature search, including information from other species.

## Results

### Genetic Parameters and Estimated Breeding Values

Descriptive statistics and genetic merit for SCS in the sampled animals are presented in Table 1. About 15,000 TD observations for SCS were considered to estimate EBVs then, the DEBVs of 5,534 individuals were estimated. Heritability and repeatability estimates for SCS in the studied population were 0.045 (standard error = 0.02) and 0.40 (standard error = 0.01), respectively.

**TABLE 1:** Descriptive statistics for somatic cell score.

Variable	N	Mean	SD	CV	Min–Max
SCS	15,008	2.67	0.72	0.27	1–5.31
DEBV	5,534	–0.19	0.52	2.75	–5.59 to 4.16

**SCS, somatic cell score; DEBV, deregressed breeding value; N, number of records; SD, standard deviation; CV, coefficient of variation; Min-Max, minimum and maximum values.**

### Quality Control for Genotyping Data

Among the 54,241 SNPs, 7,414 SNPs are located on sex chromosomes and thus were withdrawn from the analysis. A total of 3,999 SNPs were removed due to genotype rate <0.05, 2,037 SNPs due to minor allele frequency < 0.02 and 3,651 SNP due to Hardy-Weinberg disequilibrium (*P* < 10^–6^). Moreover, 12 individuals were also excluded for a low (<95%) call rate. Then, after quality control, we considered a total of 37,140 SNPs and 464 individuals for further analyses.

### Genome-Wide Association Analyses

**FIGURE 1:**
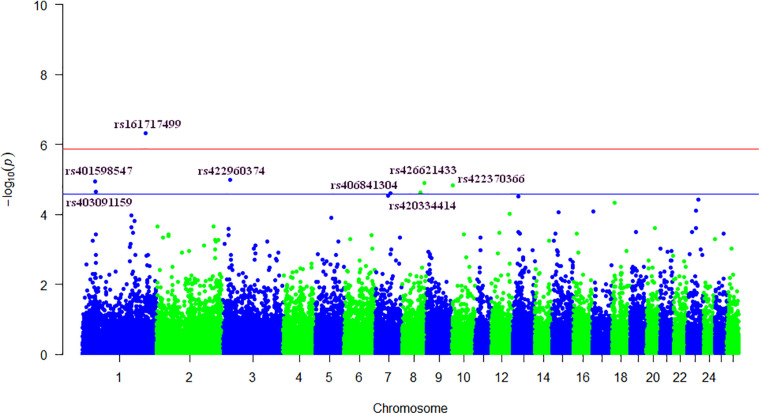
Genome-wide plot of −log10(*P*-values) for association of SNPs with somatic cell score. Blue and red lines represent suggestive [−log10(*P*-values) > 4.57] and genome-wide thresholds [−log10(*P*-values) > 5.87], respectively.

In total we detected eight significant SNPs for SCS, and among these, only one marker reached the genome-wide significant threshold (*P* < 4.72 × 10^–7^). The details of these SNPs including *P*-values, the positions on *Ovis aries* v4.0 genome assembly, the chromosomes and the closest known genes are given in Table 2. Manhattan plot, showing the profiles of the *P*-values [in terms of –log(P)] of all tested SNPs, is showed in Figure 1. The QQ-plot in Supplementary Figure S1 shows the observed and expected *P*-values of the GWAS for SCS. The genomic inflation factor (lambda) was lower than one indicating some population stratification. However, departure from this line is also expected for a really polygenic trait, as many causal SNPs may not yet have reached genome-wide significance owing to a lack of power (Power et al., 2017). The results for the MDS showed that the bulk of the samples were not separated by the first dimension, indicating a lack of substructure (Supplementary Figure S2). The eight SNPs were located on five different chromosomes: three SNPs on OAR1, one SNP on OAR3, one SNP on OAR7, two SNPs on OAR8 and one SNP on OAR10. Considering the range of ±250 kb surrounding each significant SNP, a total of 34 genes (Table 2) were found. The most significant SNP (rs161717499) was located within the coding region of the Stress Associated Endoplasmic Reticulum Protein 1 (SERP1) gene on OAR1.

**TABLE 2:** Single nucleotide polymorphisms (SNPs) significantly associated with somatic cell score at genome-wide (*P* < 1.34 × 10^–6^) and suggestive (*P* < 2.69 × 10^–5^) thresholds.

OAR	SNP	Position (bp)	−log10 (*p*-value)	Genes
1	rs401598547	46,865,607	4.94	NEGR1
1	rs403091159	49,692,787	4.65	LRRIQ3, LOC105605157, FPGT, LOC101120030
1	rs161717499	235,497,703	6.33	SIAH2, ERICH6, LOC101119269, EIF2A, SERP1, TSC22D2, TRNAR-UCG
3	rs422960374	24,797,321	4.99	FAM49A, TRNAC-GCA
7	rs406841304	57,592,284	4.60	ATP8B4, LOC105607291, DTWD1, FAM227B, FGF7
8	rs420334414	67,510,451	4.63	HIVEP2, AIG1, ADAT2
8	rs426621433	82,781,340	4.90	SOD2, WTAP, ACAT2, TCP1, MRPL18, PNLDC1, MAS1, IGF2R, LOC106991323, LOC106991303, SLC22A1, SLC22A2
10	rs422370366	4,119,025	4.82	–

**OAR, Ovis aries chromosome; Genes, the closest genes to the significant SNP found within ± 250 kb region surrounding it.**

**FIGURE 2:**
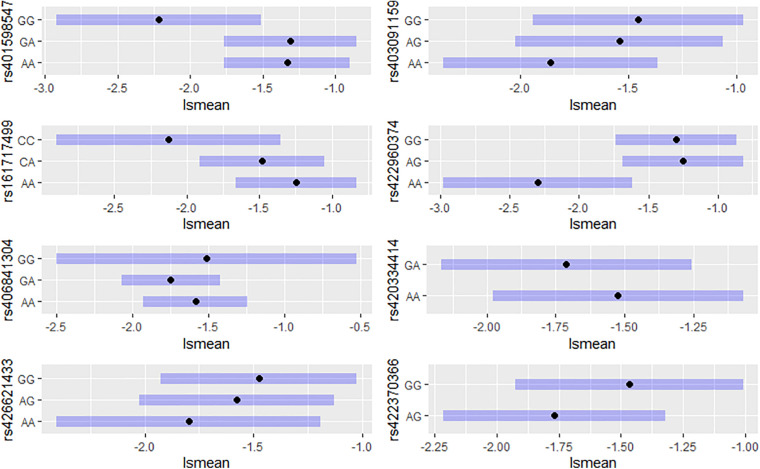
Least squares means (at 95% CI) of DEBV for the three genotypes affecting the trait of significant SNP detected from GWAS study.

For each of the eight significant SNPs, we calculated the LSM of the DEBV for the three genotypes affecting the trait to investigate their genetic contribution (Figure 2). Five out of the eight above reported SNPs (rs401598547, rs403091159, rs161717499, rs422960374, rs426621433) reached the significance threshold (*P* < 0.05). Individuals with homozygous genotypes GG for rs401598547, CC for rs161717499 and AA for rs403091159, rs422960374, and rs426621433, showed lower somatic cells content among all three genotypes (Figure 2). After checking on SheepQTLdb tool, no one of the eight detected SNPs was located within a known QTLs related at SCS or mastitis.

## Discussion

Mastitis is still one of the greatest problems affecting commercial milk production. However genetic evaluation of mastitis is particularly difficult because of the low heritability and the categorical nature of the trait. As a consequence, SCC has been promoted as an indirect method of predicting mammary infections due to the positive correlation between these two traits (Boettcher, 2005). It is worth to mention that collecting information on SCC is easier, cheaper, and less time demanding for farmers compared to use bacteriological status as direct measure of mastitis (Riggio, 2012). In this study we estimated the breeding value for SCS and identified the genomic regions putatively involved in mastitis resistance in the local economically important Valle del Belice dairy sheep breed.

The mean SCS (Table 1) was lower than those reported in previous studies (Riggio et al., 2010; Tolone et al., 2013) in the same breed, and by Ariznabarreta et al. (2002) in Churra sheep and Leitner et al. (2003) in Isaraeli-Assaf and Awassi sheep. These differences in SCS in the Valle del Belice breed were due to different sampled population. The heritability estimate for SCS in this study falls within the range (0.04–0.16) reported in literature for sheep (e.g., Barillet et al., 2001; Hamann et al., 2004; Tolone et al., 2013).

In this study, DEBVs of the SCS were used as trait scores for the association analysis. The estimated breeding values (EBVs) were generally used (like as pseudo-phenotypes) to perform the GWAS. Although EBVs have been used as dependent variables in GWAS (Johnston et al., 2011; Becker et al., 2013), this approach gave high false positive rate (Ekine et al., 2014). Consequences of using EBVs include varying levels of precision and “shrinkage effect” among the values used to represent phenotypes of different individuals, a reduction in the sample variance of the phenotypes, and double-counting of information from relatives (Garrick et al., 2009; Ostersen et al., 2011). The DEBV make good use of available information from genotyped animals as well as from their relatives, which can appropriately avoid bias introduced by simply pooling or averaging data information and account for heterogeneous variance (Garrick et al., 2009).

A total of eight SNPs were found to be significantly associated with SCS in Valle del Belice sheep. For five significant SNPs, results suggested that individuals with the GG genotypes at rs401598547, CC at rs161717499, and AA at rs403091159, rs422960374, and rs426621433, could be selected to reduce the somatic cells content in milk, although these genotypes had a low frequency in the breed. The lack of selection pressure in Valle del Belice dairy sheep may also contribute to the low frequency of the favorable alleles and genotypes. Therefore, the effect of these alleles for somatic cells content trait should be verified in a larger population or by testing them in an independent sample.

The most significant SNP associated with SCS was located in the intronic region of *SERP1*. This gene encodes the stress-associated endoplasmic reticulum protein 1 and was associated with immune system (Moravčíková et al., 2018). Another relevant gene close to the most significant SNP was *SIAH2*, involved to apoptosis and programmed cell death (Crisà et al., 2016). On the same chromosome, the other two markers were close to *NEGR1*, a gene involved with medium white blood cell count (a leukocyte trait) in Yak (Ma et al., 2019), *LRRIQ3* related to the innate immune system upon recognition of pathogens (Pablo-Maiso et al., 2018) and *FPGT*, which is part of the L-fucose pathway, a key sugar in complex carbohydrates involved in cell-to-cell recognition, inflammation, and immune processes (Becker and Lowe, 2003). As above reported, mastitis is a persistent, inflammatory response of mammary tissue attributed to intramammary invasion of a mastitis-causing pathogen. Therefore, according to their role and function, these aforementioned genes can be considered as candidate involved in mastitis resistance and SCS. The SNP rs422960374 on OAR03, was close (∼70 Kb) to the *FAM49A* gene. Marete et al. (2018), in a GWAS for milking speed in French Holstein cows, reported the *FAM49A* as candidate gene for this trait. This gene was also associated to rear udder height in Holstein cattle (Gonzalez et al., 2020). The genetic correlation between the SCS and udder attachment in sheep was observed by Casu et al. (2010); De la Fuente et al. (1996) reported the indirect selection for subclinical mastitis resistance due to the inclusion of udder morphology traits in selection objectives. Moreover, Gutiérrez-Gil et al. (2018) suggested that sheep with udders and high degree of suspension or shallow udders close to the abdominal wall should be associated to lower SCS. Despite specific functions of this gene are not known yet, all the aforementioned aspects suggested a possible involving of *FAM49A* gene in our trait. Similarly, on OAR7, near to SNP rs406841304, two close genes are related with udder conformation (*FAM227B*) (Scienski et al., 2019) and with epithelial cell proliferation and differentiation (*FGF*7) (Bazer and Slayden, 2008; Yang et al., 2020), suggesting their possible role in the epithelial mammary cell proliferation. Moreover, the *FGF7* has been reported as putative target gene in bovine mammary tissue infected with *Streptococcus uberis* (Naeem et al., 2012). Another significant SNP was located on OAR8 (rs426621433) at position 82,781,340 bp. This SNP mapped within a QTL for SCC (81.4–83.5 Mb) reported in a commercial French dairy sheep population (Rupp et al., 2015), and near a QTL for SCC (ID number 160869) on OAR 8 (80.5–80.6 Mb) in Churra sheep. Among the closest annotated genes in the region of ±250 kb surrounding it, the *SOD2* gene seems to be the most plausible candidate affecting the SCS. In fact, the expression of SOD2 at mRNA and protein levels has been reported up-regulated in the mammary glands of ewes with clinical mastitis compared to healthy ewes (Gao et al., 2019). Mitterhuemer et al. (2010) showed an increase of SOD2 gene level in mammary tissue from mastitis cows inoculated with *E. coli* 24 h after infection as compared to controls. Finally, another candidate gene mapped near SNP rs426621433 on OAR8, was *IGF2R*, with a crucial role for the regulation of cell proliferation, growth, differentiation and survival, and associated with milk production traits. In fact, Dehoff et al. (1998) showed that lactation in the bovine mammary gland is associated with increased *IGF2R* concentration.

## Conclusion

In this study, we estimated the breeding value for SCS in Valle del Belice sheep. DEBVs of the SCS were used as trait scores for the association analysis. Several candidate genes associated with SCS were identified related to immunity system and udder conformation. These candidate genes provide valuable information for future functional characterization. Therefore, our results may contribute to increase knowledge on the role the genes play in the genetically determined mechanisms involved in mastitis in sheep. The results can help improving the competitiveness of the local Valle del Belìce breed, through the development of genetic improvement programs directed toward reducing the incidence of mastitis, also considering the udder conformation into selection objectives, with planned mating between subjects carrying favorable alleles. Anyway, further studies considering a higher sample size or independent population will be needed to confirm our results.

## Data Availability Statement

The datasets presented in this study can be found in online repositories. The names of the repository/repositories and accession number(s) can be found below: https://figshare.com/ articles/dataset/GWAS_for_somatic_cell_counts_in_sheep/1379 7851.

## Ethics Statement

Ethical review and approval was not required for the animal study because the procedures involving animal sample collection followed the recommendation of directive 2010/63/EU. Sampling was carried out by trained veterinarians within the frame of vaccination Campaigns, hence no permission from the animal research ethics committee was necessary.

## Author Contributions

MT and AS: conception of the work. AM, RD, and MT: contributed to the data acquisition. MT, AS, and AM: data analysis. AS, MT, SM, and BP: results interpretation. MT, SM, and AM: drafting the article. MT, SM, MS, and AS: critical revision of the article. AS, AM, SM, MS, RD, BP, and MT: final approval of the version to be published. All authors contributed to the article and approved the submitted version.

## Conflict of Interest

The authors declare that the research was conducted in the absence of any commercial or financial relationships that could be construed as a potential conflict of interest.

## Footnotes

1 https://www.ncbi.nlm.nih.gov/genome/?term=ovis+aries

2 http://www.r-project.org

3 https://www.ncbi.nlm.nih.gov/genome/gdv/browser/genome/?id=GCF_000298735.2

4 http://www.animalgenome.org/QTLdb
