# Supplementary figures and images for: Genome-Wide Association Study Identifies New Candidate Markers for Somatic Cells Score in a Local Dairy Sheep
Source: Front Genet. 2021 Mar 22;12:643531. doi: 10.3389/fgene.2021.643531 (PMC8019815; doi:10.3389/fgene.2021.643531)

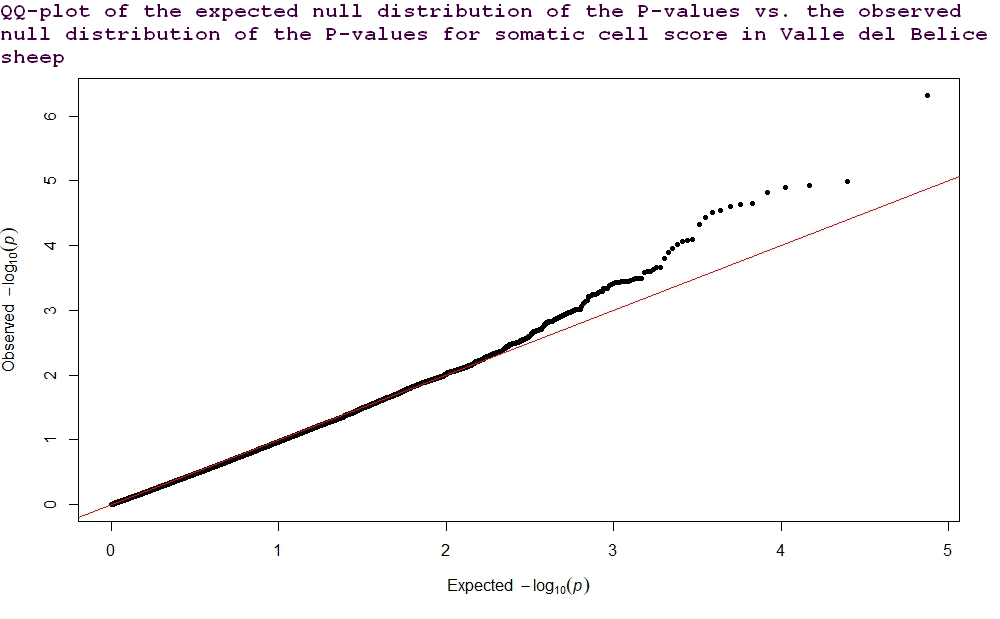

Supplement: Supplementary file 1 [file Image_1.TIF]

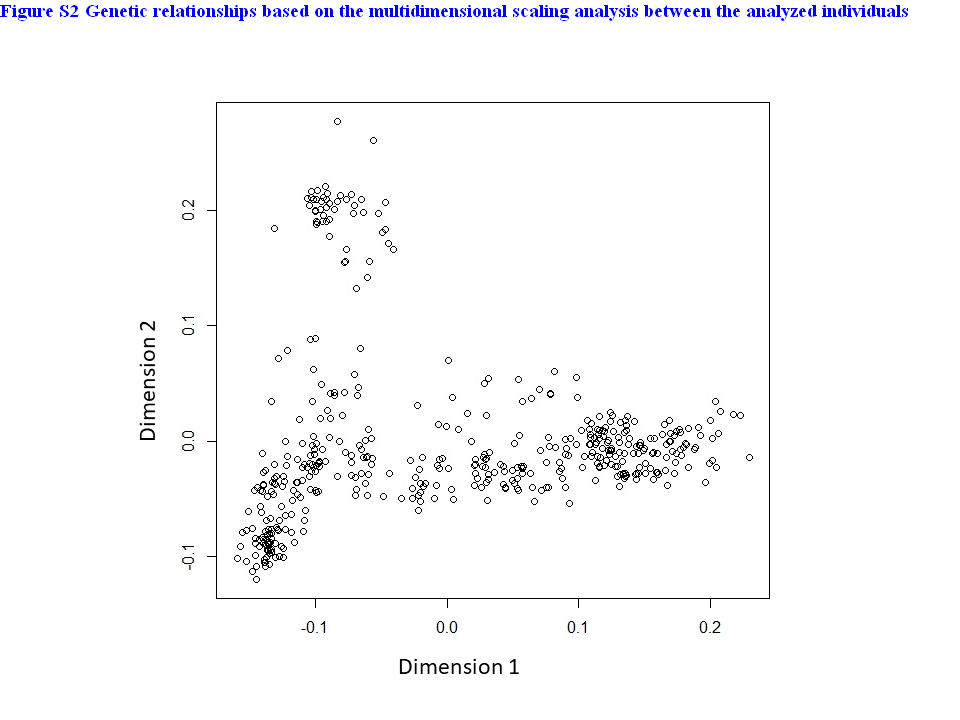

Supplement: Supplementary file 2 [file Image_2.TIF]
